# The Inflammatory-Oxidative Axis in Lung Cancer: A Comprehensive Review of Serum C-Reactive Protein, Nitric Oxide, Superoxide Dismutase, Glutathione Reductase, and Glutathione Peroxidase Alterations

**DOI:** 10.7759/cureus.116474

**Published:** 2026-09-18

**Authors:** Sushant Dhanavade

**Affiliations:** 1 Department of Biochemistry, Krishna Institute of Medical Sciences, Krishna Vishwa Vidyapeeth (Deemed to be University), Karad, IND

**Keywords:** c-reactive protein, glutathione peroxidase, glutathione reductase, inflammation, lung cancer, nitric oxide, oxidative stress, superoxide dismutase

## Abstract

Lung cancer remains the leading cause of cancer-related mortality worldwide, accounting for approximately 1.8 million deaths annually. Despite advances in targeted therapies and immunotherapy, late diagnosis and therapeutic resistance continue to limit patient outcomes. Emerging evidence implicates systemic inflammation and oxidative stress as critical drivers of lung cancer initiation, progression, and treatment resistance. This comprehensive review synthesizes current knowledge regarding alterations in serum C-reactive protein (CRP), nitric oxide (NO), superoxide dismutase (SOD), glutathione reductase (GR), and glutathione peroxidase (GPx) in patients with lung cancer. A consistent body of literature demonstrates significantly elevated CRP and NO levels alongside markedly reduced SOD, GR, and GPx activities in treatment-naïve patients with lung cancer compared to healthy controls, indicating a profound inflammatory-oxidative imbalance. However, important heterogeneity exists across studies, with the magnitude of these alterations varying by tumor stage, histological subtype, smoking history, and the biological compartment analyzed. The absence of linear correlation between inflammatory and antioxidant markers in several studies suggests complex, non-linear interactions between these pathways rather than simple opposition. Clinically, this combined biomarker panel may aid in risk stratification, disease monitoring, and evaluation of therapeutic response, particularly when integrated into established scoring systems such as the Glasgow Prognostic Score. Major knowledge gaps persist, including a lack of large prospective longitudinal studies, the absence of validated cut-off values, and limited correlation with hard clinical endpoints. Future research should prioritize multicenter cohorts with standardized assays and paired serum-tissue analyses to translate this axis into routine oncologic practice.

## Introduction and background

Epidemiology and clinical burden of lung cancer

Lung cancer persists as the most lethal malignancy worldwide, accounting for more than 1.8 million annual deaths and representing approximately 18% of all cancer-related mortality [1]. The disease is broadly classified into two main histological categories: non-small cell lung cancer (NSCLC), which constitutes approximately 85% of cases, and small cell lung cancer (SCLC), which accounts for the remaining 15% [2]. NSCLC is further subdivided into adenocarcinoma, squamous cell carcinoma, and large cell carcinoma, each with distinct molecular profiles and clinical behaviors. Despite significant advances in diagnostic imaging, minimally invasive biopsy techniques, molecular profiling, and the development of targeted therapies (e.g., epidermal growth factor receptor (EGFR) inhibitors and anaplastic lymphoma kinase (ALK) inhibitors) and immune checkpoint inhibitors (e.g., anti-programmed death protein 1 (PD-1)/programmed death ligand 1 (PD-L1) agents), the overall five-year survival rate remains dismally low at approximately 20% for all stages combined [3]. This poor prognosis is attributed to several interrelated factors, including late presentation with advanced-stage disease, frequent and early metastasis to distant organs (brain, bone, liver, and adrenal glands), intrinsic and acquired resistance to chemotherapy and targeted agents, and the limited therapeutic options available for patients with poor performance status [4].

The global burden of lung cancer is distributed unevenly, with the highest incidence rates observed in North America, Europe, and East Asia, though low- and middle-income countries are experiencing rapidly increasing rates due to rising tobacco consumption and environmental exposures [5]. In India, lung cancer accounts for approximately 7%-10% of all cancer diagnoses, with a rising incidence among younger individuals and non-smokers, suggesting additional etiologic factors beyond tobacco, including air pollution, biomass fuel exposure, and occupational hazards [6].

The emerging paradigm of inflammation and oxidative stress in carcinogenesis

Carcinogenesis in lung cancer is a multifactorial process traditionally attributed to genetic mutations (e.g., *TP53*, *KRAS*, and *EGFR*), chronic inflammatory responses (e.g., in chronic obstructive pulmonary disease and pulmonary fibrosis), environmental carcinogens (most notably tobacco smoke containing over 60 known carcinogens), and occupational exposures (asbestos, radon, and heavy metals) [7]. However, over the past two decades, a paradigm shift has occurred, recognizing that inflammation and oxidative stress are not merely secondary consequences of malignancy but rather active drivers and facilitators of carcinogenesis, tumor maintenance, and therapeutic resistance [8].

Chronic inflammation contributes to each of the hallmark capabilities of cancer, including sustained proliferative signaling, evasion of growth suppressors, resistance to cell death, induction of angiogenesis, activation of invasion and metastasis, and avoidance of immune destruction [9]. The inflammatory tumor microenvironment is characterized by the presence of tumor-associated macrophages (TAMs), myeloid-derived suppressor cells (MDSCs), regulatory T cells (Tregs), and a plethora of pro-inflammatory cytokines such as interleukin-1 beta (IL-1β), interleukin-6 (IL-6), and tumor necrosis factor-alpha (TNF-α) [10]. These cytokines not only promote tumor cell proliferation and survival through activation of nuclear factor kappa-light-chain-enhancer of activated B cells (NF-κB) and signal transducer and activator of transcription 3 (STAT3) signaling pathways but also stimulate hepatic synthesis of acute-phase reactants, most notably C-reactive protein (CRP) [11].

Concurrently, oxidative stress -- defined as an imbalance between the excessive generation of reactive oxygen species (ROS) and reactive nitrogen species (RNS) and the capacity of the endogenous antioxidant defense system to neutralize them -- plays a critical role in lung cancer pathogenesis [12]. Malignant cells typically exhibit heightened oxidative stress due to increased metabolic rate, mitochondrial dysfunction, oncogene activation, loss of tumor suppressor function, and inflammatory cytokine signaling. ROS and RNS cause oxidative damage to DNA (resulting in 8-oxo-2'-deoxyguanosine adducts and strand breaks), lipids (leading to membrane dysfunction and generation of reactive aldehydes such as malondialdehyde and 4-hydroxynonenal), and proteins (causing enzyme inactivation and altered signaling), thereby promoting genomic instability, mutagenesis, and malignant transformation [13].

The antioxidant defense system: structure and function

The human body is equipped with an intricate and multifaceted antioxidant defense system that includes enzymatic antioxidants, non-enzymatic antioxidants, and metal-binding proteins [14]. Among the enzymatic antioxidants, three families are particularly relevant to lung cancer pathophysiology.

Superoxide dismutase (SOD) constitutes the first line of defense against ROS, catalyzing the dismutation of the superoxide anion radical (O₂•⁻) into hydrogen peroxide (H₂O₂) and molecular oxygen (O₂). Three isoforms of SOD exist in humans: SOD1 (copper-zinc SOD, cytosolic), SOD2 (manganese SOD, mitochondrial), and SOD3 (extracellular SOD) [15]. The mitochondrial isoform, SOD2, is particularly critical, given that mitochondria are the primary source of endogenous ROS production through electron transport chain leakage. Reduced SOD activity allows superoxide to accumulate, which can then react with nitric oxide (NO) to form peroxynitrite, a highly damaging species that nitrates tyrosine residues on proteins and inactivates key signaling molecules.

Glutathione peroxidase (GPx) is a selenium-dependent enzyme family (GPx1 through GPx8) that catalyzes the reduction of H₂O₂ and organic hydroperoxides (including lipid hydroperoxides) to water and corresponding alcohols, using reduced glutathione (GSH) as an electron donor [16]. GPx1 is the most abundant isoform and is ubiquitously expressed, while GPx4 plays a unique, non-redundant role in inhibiting ferroptosis, an iron-dependent form of regulated cell death characterized by the accumulation of lipid peroxides [17]. Ferroptosis has emerged as a critical pathway in cancer biology, with some tumors becoming highly dependent on GPx4 for survival, creating a therapeutic vulnerability.

Glutathione reductase (GR) is a flavoprotein that catalyzes the reduction of oxidized glutathione (GSSG) to GSH using nicotinamide adenine dinucleotide phosphate (NADPH) as a cofactor [18]. By maintaining a high GSH/GSSG ratio (typically >100:1 in healthy cells), GR ensures the continued availability of GSH for GPx and other glutathione-dependent enzymes, including glutathione S-transferases (GSTs) involved in drug detoxification. NADPH, generated primarily through the pentose phosphate pathway, is the ultimate reducing equivalent that powers this system.

Non-enzymatic antioxidants include GSH, vitamins C and E, beta-carotene, uric acid, and bilirubin. These small molecules scavenge ROS directly and serve as cofactors for enzymatic antioxidants. The coordinated action of these systems maintains cellular redox homeostasis under normal conditions.

Rationale for combined biomarker evaluation

Despite extensive research supporting the individual roles of inflammatory and oxidative biomarkers in lung cancer development and progression, the combined evaluation of both biomarker classes in the same patient cohort remains surprisingly underexplored, particularly in regional populations [19]. Most previous studies have focused either on inflammatory markers (CRP and cytokines) or on oxidative stress markers (SOD, GPx, and malondialdehyde), but rarely both simultaneously. This fragmented approach limits our understanding of how inflammation and oxidative stress interact in the systemic circulation of patients with lung cancer.

Furthermore, the relationship between these two systems is theoretically bidirectional. Inflammation generates ROS through NADPH oxidase activation in phagocytes, while oxidative stress can activate NF-κB and other pro-inflammatory transcription factors through the modification of redox-sensitive cysteine residues in signaling proteins [20]. A comprehensive review that synthesizes findings across multiple studies and critically evaluates areas of agreement, inconsistency, and knowledge gaps is therefore timely and necessary.

Given the fragmented literature on the simultaneous evaluation of inflammatory and oxidative biomarkers in lung cancer, this comprehensive review aims to: (i) synthesize current evidence regarding serum alterations in CRP, NO, SOD, GR, and GPx in patients with lung cancer; (ii) critically evaluate areas of agreement, inconsistency, and knowledge gaps across studies; (iii) discuss the clinical and translational implications of this combined biomarker panel; and (iv) propose directions for future research, including the need for prospective multicenter cohorts and standardized assays. By integrating findings from both inflammatory and antioxidant axes, this review seeks to provide a unified framework for understanding systemic redox imbalance in lung cancer and to guide its potential translation into oncologic practice.

## Review

This review synthesizes findings qualitatively. No formal meta-analysis, quantitative pooling, or heterogeneity statistics were performed because the included studies vary substantially in design, assays, and populations. Statistical values reported are those of the original studies and are presented for descriptive purposes only.

Literature search strategy

A systematic literature search was performed using PubMed, Scopus, and Web of Science databases from inception to April 2026. Search terms included combinations of: ‘lung cancer’ OR ‘NSCLC’ OR ‘SCLC’ AND ‘C-reactive protein’ OR ‘CRP’ OR ‘nitric oxide’ OR ‘NO’ OR ‘superoxide dismutase’ OR ‘SOD’ OR ‘glutathione reductase’ OR ‘GR’ OR ‘glutathione peroxidase’ OR ‘GPx’ OR ‘GPX4’ OR ‘ferroptosis’ OR ‘oxidative stress’ OR ‘inflammation.’ Additional hand-searching of reference lists of included articles and relevant reviews was performed. Only peer-reviewed original research and systematic reviews/meta-analyses published in English were included. Case reports, editorials, and conference abstracts were excluded.

Inflammatory markers in lung cancer: CRP and NO

CRP: Biology and Clinical Utility

CRP is a pentameric acute-phase reactant synthesized by hepatocytes primarily under the stimulation of IL-6, with additional contributions from IL-1β and TNF-α [21]. Under normal conditions, serum CRP levels are typically below 5 mg/L. In the setting of chronic inflammation, including the tumor microenvironment of lung cancer, IL-6 produced by tumor cells, TAMs, and stromal fibroblasts drives sustained CRP elevation. The biological functions of CRP include activation of the complement system via the C1q complex, opsonization of cellular debris, and facilitation of phagocytosis, though its precise role in cancer progression remains incompletely understood.

A large body of observational studies has consistently demonstrated elevated serum CRP in patients with lung cancer compared with healthy controls. A meta-analysis by Lim and colleagues, encompassing over 5,000 patients with NSCLC, concluded that elevated pretreatment CRP (typically defined as >10 mg/L) is independently associated with worse overall survival (HR: 1.78, 95% CI: 1.45-2.18) and progression-free survival [22]. Importantly, this association persists across histological subtypes and stages, suggesting that CRP reflects a fundamental aspect of tumor biology rather than a stage-specific phenomenon.

The prognostic value of CRP is sufficiently robust that it has been incorporated into several clinical scoring systems. The Glasgow Prognostic Score (GPS), which combines CRP (>10 mg/L) and albumin (<35 g/L), and the modified GPS (mGPS), which prioritizes CRP elevation regardless of albumin status, have been validated in multiple independent cohorts of patients with lung cancer [23]. Patients with an mGPS of 2 (elevated CRP with hypoalbuminemia) have consistently demonstrated significantly worse survival compared with those with an mGPS of 0 (normal CRP), with the prognostic impact independent of stage, performance status, and treatment received.

More recently, CRP has emerged as a potential predictor of response to immune checkpoint inhibitors. Several retrospective studies have reported that elevated baseline CRP is associated with poor response to anti-PD-1/PD-L1 agents, possibly reflecting an immunosuppressive tumor microenvironment characterized by MDSC infiltration and reduced T-cell functionality [24]. Furthermore, normalization of CRP during treatment (i.e., a decrease to within the normal range) has been associated with improved outcomes, suggesting that CRP dynamics may serve as an early marker of treatment efficacy. However, prospective validation in large cohorts is needed before CRP can be recommended for treatment selection.

NO: The Double-Edged Sword

NO is a gaseous signaling molecule synthesized from L-arginine by three isoforms of NO synthase (NOS): neuronal NOS (nNOS or NOS1), inducible NOS (iNOS or NOS2), and endothelial NOS (eNOS or NOS3) [25]. Under physiological conditions, low (picomolar to nanomolar) concentrations of NO produced by eNOS and nNOS maintain vascular tone, regulate neurotransmission, and mediate immune defense through S-nitrosylation of specific protein targets.

In the tumor microenvironment, however, iNOS is frequently overexpressed in both tumor cells and infiltrating inflammatory cells, leading to sustained production of high (micromolar) concentrations of NO [26]. At these elevated levels, NO exerts pro-tumorigenic effects through multiple mechanisms. First, NO can directly damage DNA through deamination reactions (converting cytosine to uracil and adenine to hypoxanthine) and nitrosation reactions, generating mutagenic lesions that drive genomic instability. Second, NO inhibits apoptosis by S-nitrosylating and inactivating key caspases (particularly caspase-3 and caspase-8) and by upregulating anti-apoptotic proteins such as Bcl-2. Third, NO promotes angiogenesis through upregulation of vascular endothelial growth factor and stabilization of hypoxia-inducible factor-1 alpha. Fourth, NO facilitates metastasis by activating matrix metalloproteinases and inducing epithelial-mesenchymal transition [27].

The concentration-dependent duality of NO function is critical to interpret clinical studies. Several case-control studies have reported significantly elevated serum or plasma nitrite/nitrate (NOx, the stable oxidation products of NO) in patients with lung cancer compared with healthy controls, with levels correlating with tumor stage, nodal involvement, and poor survival. Jian-Hua and colleagues reported a mean plasma NOx level of 32.4 μmol/L in NSCLC patients vs. 18.7 μmol/L in controls, with the highest levels observed in stage IV disease [28]. However, some studies have reported lower NO levels in certain patient subsets, possibly reflecting differential iNOS expression or consumption of NO by superoxide to form peroxynitrite.

A representative case-control study comparing treatment-naïve patients with lung cancer to age- and sex-matched healthy controls (Table 1) [29] illustrates the magnitude of these alterations, showing a fivefold elevation in CRP (22.67 vs. 4.60 mg/dL) and a 62% increase in NO (32.30 vs. 19.90 μmol/L). These values are consistent with the pro-tumorigenic role of excessive inflammation and RNS generation, though the absence of iNOS immunohistochemistry or distinction between NO derived from tumor vs. inflammatory cells remains a limitation common to most serum-based studies.

**Table 1:** Serum inflammatory and antioxidant biomarkers in lung cancer patients vs. healthy controls Data are presented as mean ± SD. Lung cancer patients (n=50) were treatment-naïve at the time of sample collection. Healthy controls (n=50) were age- and sex-matched. Statistical comparison was performed using an independent samples t-test. The percentage change represents the relative difference between lung cancer patients and healthy controls, calculated as Group I mean - Group II mean/Group II mean × 100. A positive percentage indicates an increase and a negative percentage indicates a decrease in lung cancer patients compared to controls. Data source: [29] CRP, C-reactive protein; GPx, glutathione peroxidase; GR, glutathione reductase; GSH, reduced glutathione; NADPH, nicotinamide adenine dinucleotide phosphate; NO, nitric oxide; SD, standard deviation; SOD, superoxide dismutase.

Parameter (unit)	Lung cancer patients (mean ± SD)	Healthy controls (mean ± SD)	Change	p-value
CRP (mg/dL)	22.67 ± 6.20	4.60 ± 3.50	↑ 5-fold	<0.001
NO (μmol/L)	32.30 ± 7.50	19.90 ± 5.20	↑ 62%	<0.001
SOD (U/mL)	2.86 ± 0.16	5.11 ± 0.60	↓ 44%	<0.001
GR (mM NADPH/min/g)	3.25 ± 0.34	9.76 ± 0.43	↓ 67%	<0.001
GPx (mM GSH/min/mg)	16.52 ± 1.25	34.96 ± 6.29	↓ 53%	<0.001

Antioxidant enzyme alterations in lung cancer

SOD: Depleted First-Line Defense

The marked reduction in SOD activity in patients with lung cancer compared with healthy controls, as demonstrated in multiple studies including the representative data shown in Table 1 (44% reduction, from 5.11 to 2.86 U/mL), suggests a compromised capacity to neutralize superoxide radicals [29-31]. Several mechanisms may account for this depletion.

First, chronic oxidative stress in the tumor microenvironment may exhaust antioxidant enzymes faster than they can be regenerated. Superoxide production is elevated in lung cancer due to mitochondrial dysfunction, NADPH oxidase activation, and inflammatory cell infiltration. Under normal conditions, SOD is recycled efficiently, but a sustained high flux of superoxide can lead to irreversible inactivation, particularly of the mitochondrial SOD2 isoform, which is vulnerable to nitration and oxidation [15].

Second, transcriptional and post-transcriptional downregulation of SOD expression may occur. The nuclear factor erythroid 2-related factor 2 (Nrf2) pathway is the master regulator of antioxidant gene expression, controlling SOD1, SOD2, catalase, and numerous other cytoprotective genes. While some cancers exhibit constitutive Nrf2 activation (due to KEAP1 mutations or promoter hypermethylation) that confers treatment resistance, others demonstrate impaired Nrf2 signaling, leading to reduced expression of SOD and other antioxidants [32]. The net effect appears to be context-dependent, with some studies reporting elevated SOD in tumor tissue as an adaptive response and others reporting reduced SOD in serum as evidence of systemic depletion.

Third, post-translational modifications directly inactivate SOD enzymes. Peroxynitrite, formed from the rapid reaction of NO with superoxide (a reaction that occurs at near diffusion-limited rates), can nitrate tyrosine residues on SOD1 and SOD2, reducing their catalytic activity by up to 80% [33]. This mechanism creates a vicious cycle: elevated NO and superoxide generate peroxynitrite, which inactivates SOD, leading to further superoxide accumulation and more peroxynitrite formation.

Importantly, the direction and magnitude of SOD alterations vary considerably across studies. Skorska and colleagues reported that low serum total SOD activity was an independent predictor of all-cause mortality in a cohort of 120 patients with lung cancer, with an HR of 2.34 for patients in the lowest tertile [31]. However, other investigators have reported elevated SOD activity in tumor tissue itself, particularly in early-stage disease, suggesting that tissue and systemic compartments may show opposite patterns. This discrepancy highlights the importance of distinguishing between serum measurements (reflecting systemic redox status) and tissue measurements (reflecting local adaptive responses).

*GPx* *: Linking Redox Balance to Ferroptosis*

The reduction in GPx activity observed in patients with lung cancer (Table 1 shows a 53% reduction, from 34.96 to 16.52 mM GSH/min/mg) is clinically significant for several reasons. GPx, particularly the GPx1 isoform, is the primary enzyme responsible for reducing H₂O₂ to water, thereby preventing the formation of highly reactive hydroxyl radicals through the Fenton reaction [16]. When GPx activity is compromised, H₂O₂ accumulates and can react with ferrous iron to generate hydroxyl radicals, which cause indiscriminate damage to DNA, proteins, and lipids.

Of particular note in the context of lung cancer is the role of GPx4 in inhibiting ferroptosis. Ferroptosis is an iron-dependent, non-apoptotic form of regulated cell death characterized by the accumulation of lipid peroxides to lethal levels [17]. GPx4 is the only enzyme capable of reducing lipid hydroperoxides within cellular membranes; when GPx4 is inhibited or depleted, lipid peroxides accumulate, iron-dependent oxidative damage ensues, and ferroptosis is triggered. Emerging evidence indicates that some cancer cells, including certain subsets of lung cancer, become highly dependent on GPx4 for survival and are therefore exquisitely sensitive to ferroptosis-inducing agents [34].

The observation of reduced GPx activity in the serum of patients with lung cancer may appear paradoxical: if GPx is essential for survival, why would cancer cells downregulate it? Several explanations are plausible. First, serum GPx activity primarily reflects GPx3 (the extracellular isoform), which is distinct from the intracellular GPx1 and GPx4 isoforms that are critical for cancer cell survival. Second, cancer cells may upregulate alternative antioxidant systems, such as the thioredoxin system, to compensate for reduced GPx activity. Third, the serum measurement represents systemic spillover rather than tumor-specific activity; the tumor itself may maintain adequate GPx activity while other tissues become depleted.

GR: Maintaining the GSH Pool

GR maintains the GSH pool by reducing GSSG back to GSH at the expense of NADPH. The profound reduction in GR activity shown in Table 1 (67% reduction, from 9.76 to 3.25 mM NADPH/min/g) would be expected to dramatically impair the GSH/GSSG ratio, compromising not only GPx function but also the function of GSTs involved in detoxification of chemotherapy drugs [18].

Low GR activity in patients with lung cancer has been reported in multiple independent studies. A study by Huang measured erythrocyte GR activity in 75 NSCLC patients and 50 healthy controls, finding significantly lower activity in patients (3.18 ± 0.41 vs. 9.52 ± 0.56 U/g Hb), with the lowest levels observed in patients who subsequently showed poor response to platinum-based chemotherapy [30]. This finding raises the possibility that GR activity may predict treatment response, though prospective validation is needed.

The mechanisms underlying GR depletion likely parallel those for SOD and GPx: exhaustion due to chronic oxidative stress, transcriptional downregulation, and post-translational inactivation. Additionally, GR requires NADPH as a cofactor, and the pentose phosphate pathway (the primary source of NADPH) may be dysregulated in cancer, further compromising GR activity [18].

The Absence of Linear Correlation: Complex Interplay

An unexpected but informative finding from several studies, including the representative study shown in Table 1, is the absence of significant linear correlation between inflammatory markers (CRP and NO) and antioxidant enzymes (SOD, GR, and GPx) [29]. This does not imply that inflammation and oxidative stress are independent processes; rather, it suggests a complex, non-linear interplay that is not captured by simple correlation analysis.

Several explanations are plausible. First, different regulatory networks govern inflammation and antioxidant responses. Inflammation is primarily driven by the NF-κB pathway, while antioxidant responses are regulated by Nrf2. These pathways can operate in parallel rather than in direct linear opposition, and they can be differentially activated depending on the specific stressors present [32]. Second, the relationship may be non-linear, with antioxidant enzymes initially upregulated in response to inflammation (as an adaptive response) but subsequently depleted as chronic inflammation persists. A cross-sectional study measuring only a single time point would miss this temporal dynamic. Third, serum levels may not capture localized redox interactions within the tumor microenvironment. Significant correlations might exist within tumor tissue that are not reflected in systemic circulation due to dilution, compartmentalization, or differential clearance.

Thus, clinicians should interpret these biomarkers as complementary rather than interchangeable. A panel approach -- measuring both inflammatory and antioxidant markers -- likely provides more comprehensive information than any single biomarker alone. The combination of high CRP/low albumin (elevated mGPS) with low SOD/GPx may identify a particularly high-risk subgroup with both inflammatory activation and compromised antioxidant reserve.

Clinical and translational implications

The biomarker panel comprising CRP, NO, SOD, GR, and GPx has several potential clinical applications, though it is important to recognize that most have not yet been prospectively validated.

Risk Stratification

Patients with high CRP/NO and low SOD/GR/GPx may represent a subgroup with particularly aggressive disease and poor prognosis. This could guide the intensity of initial therapy, with such patients potentially benefitting from more aggressive multimodality treatment or inclusion in clinical trials of novel agents [35].

Treatment Monitoring

Serial measurements of these biomarkers during treatment could track systemic response to chemotherapy, radiotherapy, immunotherapy, or targeted agents. For instance, rising antioxidant enzyme levels during treatment might indicate restoration of redox balance and a favorable prognosis, while persistently low levels might herald treatment resistance or disease progression. Normalization of CRP during immunotherapy has already been associated with improved outcomes [24].

Therapeutic Targeting

Understanding the inflammatory-oxidative axis opens avenues for therapeutic intervention. Strategies under investigation include Nrf2 activators (e.g., sulforaphane and bardoxolone methyl) to restore antioxidant capacity, pro-oxidant drugs (e.g., elesclomol and piperlongumine) to enhance oxidative stress selectively in cancer cells, inhibitors of iNOS (e.g., aminoguanidine and L-NIL) to reduce NO production, and GPx4 inhibitors (e.g., RSL3 and ML162) to induce ferroptosis in GPx4-dependent tumors [34,36].

Comparison with existing literature and areas of contradiction

While the general pattern of elevated CRP/NO and reduced SOD/GR/GPx in patients with lung cancer is consistent across most studies, important contradictions merit discussion. Some studies have reported increased levels of certain antioxidant enzymes in tumor tissue or serum as an adaptive response to oxidative stress [37]. For example, Saydam and colleagues reported significantly higher SOD activity in lung tumor tissue than adjacent non-malignant tissue, particularly in squamous cell carcinoma [38]. This discrepancy may be attributed to several factors.

First, the compartment being analyzed matters critically. Tumor tissue is exposed to high local ROS and may upregulate antioxidant enzymes as a survival mechanism, while serum reflects systemic spillover and may show net depletion due to consumption and impaired synthesis in non-tumor tissues. Second, the timing of sample collection relative to treatment is crucial. Treatment-naïve patients (as in the representative study shown in Table 1) show different profiles than patients who have received chemotherapy or radiotherapy, which themselves induce oxidative stress. Third, histological subtype differences exist, with squamous cell carcinoma (more strongly associated with smoking) showing different biomarker profiles than adenocarcinoma (more common in never smokers) [3]. Fourth, genetic polymorphisms in antioxidant enzyme genes (e.g., SOD2 Val16Ala, GPx1 Pro198Leu) may influence baseline enzyme activities and cancer risk, though this area requires further investigation.

Limitations of the current literature and future directions

Despite the growing body of evidence supporting the inflammatory-oxidative axis in lung cancer, the literature suffers from several methodological limitations that constrain clinical translation. Importantly, all studies reviewed herein are observational in nature, and causation cannot be inferred from the reported associations between biomarker alterations and clinical outcomes. First, the majority of studies are cross-sectional with modest sample sizes (typically fewer than 100 patients per group) from single centers, establishing association but not causation. Second, marked heterogeneity exists in biomarker assays (spectrophotometric vs. enzyme-linked immunosorbent assay vs. multiplex), specimen types (serum vs. plasma vs. tissue), and reference ranges, precluding meta-analysis or definition of validated cut-offs. Third, most studies lack detailed clinicopathological annotation -- specifically tumor stage (I-IV), histologic subtype, smoking pack-years, performance status, and concurrent medications -- which are critical confounders. Fourth, the absence of longitudinal sampling means we do not know whether antioxidant enzyme levels rise, fall, or fluctuate with treatment or disease progression. Fifth, very few studies have correlated serum biomarkers with intratumoral expression of Nrf2, iNOS, or GPx4, leaving the relationship between systemic and compartment-specific redox status unclear. Sixth, the sample sizes are generally insufficient for subgroup analyses by histology, stage, or treatment type.

Future investigations should prioritize prospective, multicenter cohort studies with standardized pre-analytical protocols (including time of day of blood draw, fasting status, and processing procedures), paired serum and tumor tissue collection, predefined clinical endpoints (overall survival, progression-free survival, and response to specific therapies), and serial sampling at diagnosis, during treatment, and at progression. Such studies will be essential to determine whether this biomarker panel can guide clinical decisions, such as selecting patients for Nrf2-activating or ferroptosis-inducing therapies. Additionally, the integration of these biomarkers with established clinical tools such as the mGPS should be evaluated to determine whether the addition of oxidative markers improves prognostic accuracy beyond inflammation alone.

## Conclusions

Patients with lung cancer exhibit a profound systemic imbalance characterized by elevated inflammation (increased CRP and NO) and compromised antioxidant defense (decreased SOD, GR, and GPx) compared to healthy individuals. This biomarker profile supports the paradigm of heightened oxidative stress in lung cancer and may serve as a composite signature of disease burden. The absence of linear correlation between inflammatory and antioxidant markers underscores the complex, non-linear nature of redox biology in cancer, suggesting that these biomarker classes provide complementary rather than redundant information. Clinically, this panel offers a simple, cost-effective approach to assessing systemic inflammation and oxidative stress, with potential applications in risk stratification, treatment monitoring, and therapeutic targeting. However, major knowledge gaps persist, including a lack of large prospective longitudinal studies, the absence of validated cut-off values, and limited correlation with hard clinical endpoints. Future prospective, longitudinal studies with larger, well-characterized cohorts are needed to validate prognostic and predictive utility and to integrate these biomarkers into routine oncology practice.
